# Recognition of knowledge translation practice in Canadian health sciences tenure and promotion: A content analysis of institutional policy documents

**DOI:** 10.1371/journal.pone.0276586

**Published:** 2022-11-17

**Authors:** Kathryn M. Sibley, Masood Khan, Davina Banner, S. Michelle Driedger, Heather L. Gainforth, Ian D. Graham, Katrina Plamondon

**Affiliations:** 1 Department of Community Health Sciences, University of Manitoba, Winnipeg, MB, Canada; 2 George & Fay Yee Centre for Healthcare Innovation, Winnipeg, MB, Canada; 3 School of Nursing, University of Northern British Columbia, Prince George, BC, Canada; 4 School of Health and Exercise Sciences, University of British Columbia, Kelowna, BC, Canada; 5 International Collaboration on Repair Discoveries, University of British Columbia, Vancouver, BC, Canada; 6 School of Epidemiology and Public Health, University of Ottawa, Ottawa, ON, Canada; 7 Centre for Implementation Research, Ottawa Hospital Research Institute, Ottawa, ON, Canada; 8 School of Nursing, University of British Columbia, Kelowna, BC, Canada; La Trobe University - Melbourne Campus: La Trobe University, AUSTRALIA

## Abstract

**Background and objective:**

There has been growing emphasis on increasing impacts of academic health research by integrating research findings in healthcare. The concept of knowledge translation (KT) has been widely adopted in Canada to guide this work, although lack of recognition in tenure and promotion (T&P) structures have been identified as barrier to researchers undertaking KT. Our objective was to explore how KT is considered in institutional T&P documentation in Canadian academic health sciences.

**Methods:**

We conducted content analysis of T&P documents acquired from 19 purposively sampled research-intensive or largest regional Canadian institutions in 2020–2021. We coded text for four components of KT (synthesis, dissemination, exchange, application). We identified clusters of related groups of documents interpreted together within the same institution. We summarized manifest KT content with descriptive statistics and identified latent categories related to how KT is considered in T&P documentation.

**Results:**

We acquired 89 unique documents from 17 institutions that formed 48 document clusters. Most of the 1057 text segments were categorized as dissemination (n = 851, 81%), which was included in 47 document clusters (98%). 15 document clusters (31%) included all four KT categories, while one (2%) did not have any KT categories identified. We identified two latent categories: primarily implicit recognition of KT; and an overall lack of clarity on KT.

**Conclusions:**

Our analysis of T&P documents from primarily research-intensive Canadian universities showed a lack of formal recognition for a comprehensive approach to KT and emphasis on traditional dissemination. We recommend that institutions explicitly and comprehensively consider KT in T&P and align documentation and procedures to reflect these values.

## Introduction

Integrating health research evidence into decision-making, health care practices, and policies is critical for optimizing health outcomes and societal benefit from public investment in academic health research [1, 2]. Academic traditions have long-emphasized scholarly outputs associated with the production of evidence [3–5] despite proposals to expand concepts of scholarship [6, 7]. Such outputs include peer-reviewed publications, book chapters, patents, and academic conference presentations [8, 9]. These traditional outputs remain a cornerstone of academic activity [10–12], but have minimal impact on health care practices [13, 14], policies [15], and communities [16–18].

Efforts to bring academic health research and healthcare systems together use numerous terms, such as knowledge mobilization or knowledge translation [19, 20]. In Canada, the term Knowledge Translation (KT) was widely adopted following the publication of a definition in 2000 by the national health research funding agency, the Canadian Institutes of Health Research (CIHR). This definition, which has been adopted by multiple organizations globally [21, 22], describes KT as:

“a dynamic and iterative process that includes synthesis, dissemination, exchange and ethically-sound application of knowledge to improve health, provide more effective health services and products and strengthen the health care system. This process takes place within a complex system of interactions between academic researchers and knowledge users which may vary in intensity, complexity and level of engagement depending on the nature of the research and the findings as well as the needs of the particular knowledge user.” [23]

The components of KT are defined in Table 1, column A. A knowledge user is defined as “an individual who is likely to be able to use the knowledge generated through research to make informed decisions about health policies, programs or practices” [23]. The activities involved in KT can have transformative impact beyond traditional academic boundaries because of their focus on meeting the needs of knowledge users [24, 25], but require moving beyond longstanding academic traditions towards a fundamental “re-imagining” of academic health research [26, 27]. The 2012 San Francisco Declaration on Research Assessment (DORA), with more than 20000 signatories from 148 countries, offers a glimpse of this re-imagining by calling for outputs beyond research article production and impact factors [28].

**Table 1:** Components of knowledge translation.

KT component as defined by CIHR [23]	Example activities
Synthesis: “The contextualization and integration of research findings of individual research studies within the larger body of knowledge on the topic. A synthesis must be reproducible and transparent in its methods, using quantitative and/or qualitative methods. It could take the form of a systematic review, follow the methods developed by the Cochrane Collaboration, result from a consensus conference or expert panel or synthesize qualitative or quantitative results.”	• Systematic reviews and meta-analyses • Scoping reviews • Meta-ethnography • Realist synthesis • Practice guidelines
Exchange: “The interaction between the knowledge user and the researcher, resulting in mutual learning… [It] is collaborative problem-solving between researchers and decision-makers that happens through linkage, exchange, and co-production. Effective knowledge exchange involves interaction between decision-makers and researchers and results in mutual learning through the process of planning, producing, disseminating, and applying existing or new research in decision-making.”	• Interdisciplinary collaborations • Research collaboration • Engaged scholarship • Community engagement • Partnership with media • Partnership with industry • Integrated knowledge translation
Application: “The iterative process by which knowledge is put into practice.”	• Patents • Technology transfer • Quality improvement • Implementation of programs/ resources • Change in clinical/professional practice • Policy change • Research impact
Dissemination: “Identifying the appropriate audience and tailoring the message and medium to the audience. Dissemination activities can include such things as summaries for / briefings to stakeholders, educational sessions with patients, practitioners and/or policy makers, engaging knowledge users in developing and executing dissemination/implementation plan, tools creation, and media engagement.”	• Research articles • Books and book chapters • Publishing reviews • Conference presentations • Public presentations • Invited lectures • Symposiums • Workshops • Publishing abstracts

KT can only reach transformative potential if it is actively practiced. One of many factors that may influence engagement in KT is the role of recognition for KT efforts in performance and advancement considerations in the academic system, particularly regarding academic tenure and promotion [29–31]. Academic tenure refers to a permanent appointment of university-based faculty [32]. It is considered primarily a safeguard of academic freedom, as it allows faculty the liberty to engage in research, teaching, and service without undue consequences for employment [33]. It has a major practical benefit of long-term job security. Academic promotion is a related concept that refers to advancement of faculty rank, which typically includes assistant professor, associate professor, and full professor [27]. Several studies, including research we have conducted on Canadian health researcher experiences practicing KT [34], have identified the lack of recognition in tenure and promotion considerations as a barrier to engaging in KT [31, 35–39].

In Canada, universities individually develop Tenure and Promotion (T&P) policies. These T&P policies typically include criterion standards and/ or process guidelines for application and evaluation [40], and are often structured through formalized agreements between faculty unions or associations (where they exist) and institutions. While some of these policies and documents may apply to an entire institution, focused policies or supplements may exist at the unit level. While we are aware of cases of KT activity being incorporated in T&P policies [3, 4, 8], no comprehensive analysis has explored how academic institutions account for KT in academic recognition. Existing studies have examined components of KT in Canada and internationally [41, 42]. These include a 2021 analysis of *dissemination* activities in faculties of medicine at fifteen research-intensive universities in Canada that found an emphasis on traditional indicators. T&P consideration of activities aligning with the concept of *exchange* have also been examined, most recently in 2019 by Alperin et al. [43], who analyzed 864 T&P documents from all disciplines at 129 Canadian and American universities for the extent of ‘publicness’ in promotion considerations. They found that 64% of institutions included at least one mention of public and or community engagement in research and scholarship.

Although these are useful contributions, we note: i) the clear evidence gap with respect to the study of *synthesis* and *application* (two major components of KT) considerations in T&P; and ii) the absence of consideration for the many health science disciplines which extend beyond medicine. We argue that a comprehensive and critical examination of KT considerations is important for identifying strengths and opportunities in current approaches to T&P in academic health sciences. In turn, this can help clarify policies and guidance for engaging, reporting, and evaluating KT practice in academic health sciences. As a first step, institutional T&P documentation is important and can provide some information about how KT is formally recognized in academic health sciences. At a surface (manifest) level, the specific terms, descriptions, and examples explicitly described in T&P policies may provide guidance both to faculty members when applying for T&P, and to reviewers when evaluating applications. On a more subtle, or latent level, the discourses of these documents are not mere guidelines. They set a tone and operationalize cultural norms and expectations. They codify and reinforce assumptions about what is valued and upheld as a standard of ‘excellence’, and as such, are structural drivers of the relationship between health research and society. Collectively, both manifest and latent content reflect underlying values of an academic institution by illustrating the ‘true’ picture of organizational expectations and practices [44], and accordingly may influence researcher behavior.

With this in mind, our study was guided by two research questions: How are KT activities included and described in Canadian academic health sciences institutional T&P documentation? How do KT activities included in institutional T&P documentation reflect academic institutional values and expectations for undertaking KT?

## Methods

### Study design and methodology

We used a qualitative descriptive approach [45] including a systematic search, document review, and content analysis [46, 47]. Our approach drew on established methods for evidence synthesis and the growing body of scholarly conceptualizations of KT. Recognizing the role research and KT play in optimizing health and societal well-being, our approach was consistent with our pragmatic goals of enhancing and improving recognition of KT, starting with a gaze at institutional documents that serve to profess institutional values and reinforce what ‘counts’ as scholarship. The University of Manitoba Health Research Ethics Board exempted this study from ethics approval due to the secondary nature of document review.

### Sampling

We began by purposively sampling Canadian academic institutions and identifying health sciences disciplines at each institution to include in a search of their institutional T&P documents. We selected 15 research-intensive universities [48] known as the U15 Group of Canadian Research Universities–an organized coalition of universities who conduct approximately 80% of research in Canada [49]. We also selected the largest university as defined by student enrollment from each of the four remaining provinces and territories not represented among the research-intensive universities. We identified nineteen institutions in total. Next, a research assistant searched the institutional websites of the selected universities to identify health sciences disciplines at each institution. As there is no standard definition of health sciences, and there is considerable variation in institutional organization of academic units for health sciences across Canada, we used an inclusive and iterative process. The research assistant searched for a faculty, college, or school with any of the following terms: medicine, dentistry, nursing, public health, kinesiology, rehabilitation, physiotherapy, occupational therapy, pharmacy, and health studies/ sciences, population health, and epidemiology. Additional academic units were included as they were identified if they were based within an institution’s health sciences structure (e.g., dental hygiene, optometry, nutrition). The health sciences identification strategy was verified by a second research assistant, who repeated the search and identification process for four universities. There were no disagreements between the two research assistant decisions. The remaining universities were searched by one research assistant.

### Document search and collection

For each identified health sciences academic unit at each institution, we searched for the most current institutional documents relating to T&P, which included collective agreements, T&P policy documents, guidelines, and templates. We conducted a preliminary online search and found that several institutions had T&P documents publicly available. Two research assistants independently searched the health sciences websites for five randomly-selected English-language institutions using the following terms: tenure, promotion, career advancement, career development, guidelines, by-laws, policy/ies, checklist, criteria, procedure, and process. The two research assistants obtained the same set of T&P documents, so the document search for the remaining English-language institutions were completed by one research assistant. For the one bilingual university in the sample, the research assistant obtained the English version of T&P documents. Document search for two French-language universities was carried out by a third, bilingual research assistant. The document search was carried out in September-October 2018 and updated in June-July 2020. Where documents were not publicly available, we emailed the unit to request these documents. Once documents were acquired, we reviewed each for content related to T&P evaluation criteria. We removed duplicates and those not containing content related to T&P evaluation criteria (such as procedural and administrative documents).

### Data analysis

For each relevant and unique T&P document, we tracked the institution and academic unit it applied to, website URLs, access date, if an institution had a formal faculty union, type of document (policy, guideline, template, and collective agreement), whether they were targeted specifically for health sciences or applied to the entire institution, and version date of documents. We summarized document characteristics with descriptive quantitative statistics (frequencies and proportions).

We then reviewed each document to identify all text segments related to KT. We developed a codebook in advance (S1 Appendix) to document each segment according to categories for each component in the definition of KT (synthesis, dissemination, exchange, and application) and identify KT activities noted in established literature that corresponded to each category [23, 50, 51]. Three coders (KMS, DB, MK) piloted the codebook with documents from two institutions, which resulted in refinement of the coded activities with the addition of a generic KT code for unspecified KT activities. We focused exclusively on KT activities within research criteria for T&P, not on teaching or service criteria. We included specific scholarship of teaching activities (e.g. development and integration of an educational tool) rather than generic teaching activity involving instructional teaching of academic courses. We only coded each unique KT activity once at first instance in a document. Remaining documents were coded by one research coordinator (MK) using NVivo 10 for Windows (QSR International, Melbourne) and codes were reviewed by three investigators from five Canadian universities in three provinces who were an assistant professor (HLG or KP), associate professor (DB or KMS) and full professor academic ranking (SMD or IDG) at the time of analysis. Codes were finalized when there was agreement between at least two reviewers, and we discussed any disagreements among two or more reviewers at a full team meeting until a consensus code was reached. This approach involving a trio of investigators reviewing each code provided rich rigor to analysis [52].

During data collection and coding, we identified variation in organization of documents across units and institutions. There were often multiple documents that applied to a particular unit (such as an institutional collective agreement and disciplinary guideline), suggesting to us that they formed a related set and would likely be considered collectively by an individual reader. We also determined that many of the same documents or sets of documents were used by multiple units within an institution. In response, we then identified “clusters” of documents: groups of documents used by one or more health sciences academic units at an institution. We then organized KT activity codes and categories by document cluster, and conducted manifest content analysis in a descriptive quantitative summary (frequencies and proportions) of KT activities and categories at the document cluster level.

Lastly, we conducted a latent content analysis to interpret the data and identify categories related to implications about how KT is considered in Canadian health sciences T&P documents. The principal investigator (KMS) reviewed the descriptive quantitative summary and team meeting minutes to develop draft categories. We reviewed the proposed categories via document review and discussed them at a team meeting. We revised and refined categories until we reached consensus. We kept detailed notes of meeting discussions, decisions, and analytic memos throughout analysis to support rigor.

## Results

### Search findings, document and cluster characteristics

**Fig 1:**
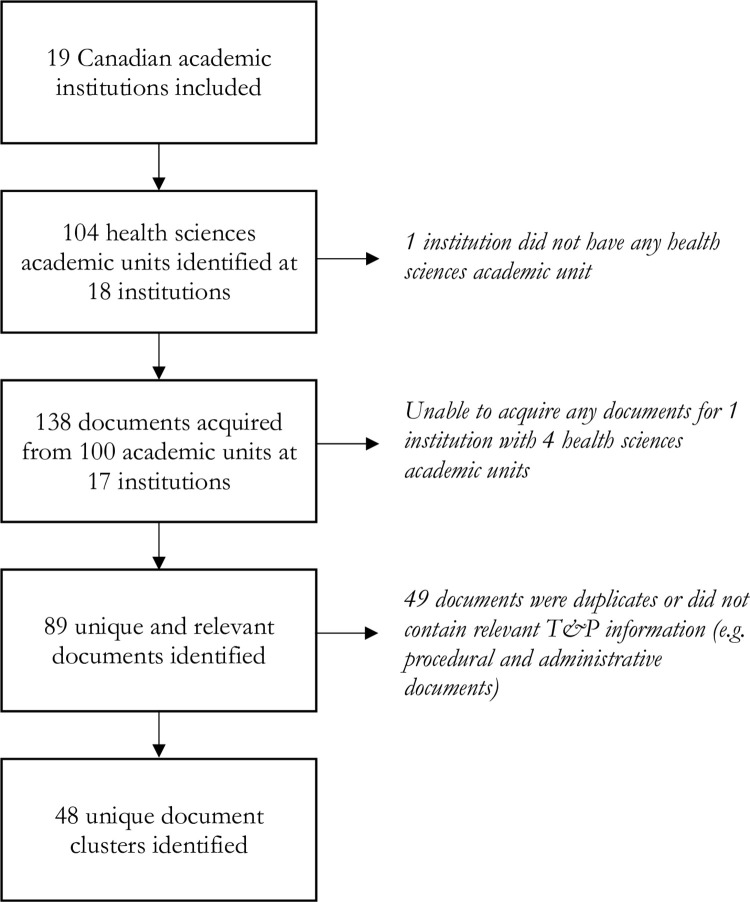
Document search flowchart.

Search results are provided in Fig 1. We identified 104 health sciences academic units across 18 of the 19 institutions (S2 Appendix). One institution did not have any health sciences academic units. We acquired 138 documents pertaining to 100 health sciences academic units from 17 institutions. Of these, 131 documents were acquired from the online search and seven were obtained from email inquiries. We were not able to acquire any documents for one institution that had four health sciences units. 13 of the 17 institutions had unionized agreements with faculty (i.e. collective agreements). Of the 138 acquired documents, we identified 89 that were unique and contained information about T&P. We identified 48 document clusters used by one or more health sciences academic units, with a range of 1–9 documents per cluster.

Characteristics of the document clusters are provided in Table 2. Most document clusters (n = 34, 71%) included guidelines. The version dates of these documents ranged from April 1980 to June 2020. All document clusters included at least one document produced between 2010–2020, and 12 included at least one document produced before 2010 (25%). Most document clusters (n = 33, 69%) had both institution-level and unit-level documents.

**Table 2:** Document cluster characteristics (n = 48).

Characteristic	Number of document clusters (%)
Document type	
Guidelines	34 (71)
Policies	31 (65)
Collective agreements	25 (52)
Templates	12 (25)
Document version date	
2010–2020	48 (100)
2000–2009	12 (25)
1990–1999	4 (8)
1980–1989	6 (12)
Unknown	11 (23)
Document level	
Institution-level only	11 (23)
Unit-level only	4 (8)
Both	33 (69)

### Manifest KT content

We identified 1057 text segments related to KT (range 0–103 segments/document cluster). Most of the segments were categorized as dissemination (n = 858, 81% of text segments) followed by application (n = 79, 7%), synthesis (n = 69, 6%), exchange (n = 32, 3%), and 19 instances of a non-specific KT category (2%). Dissemination was represented in 47 document clusters (98%), application was represented in 39 document clusters (81%), synthesis was represented in 28 document clusters (58%), and exchange was represented in just 20 document clusters (42%). Most document clusters included mention of three (n = 17, 35%) or four (n = 15, 31%) KT categories, while one (2%) did not have any KT categories identified.

**Fig 2:**
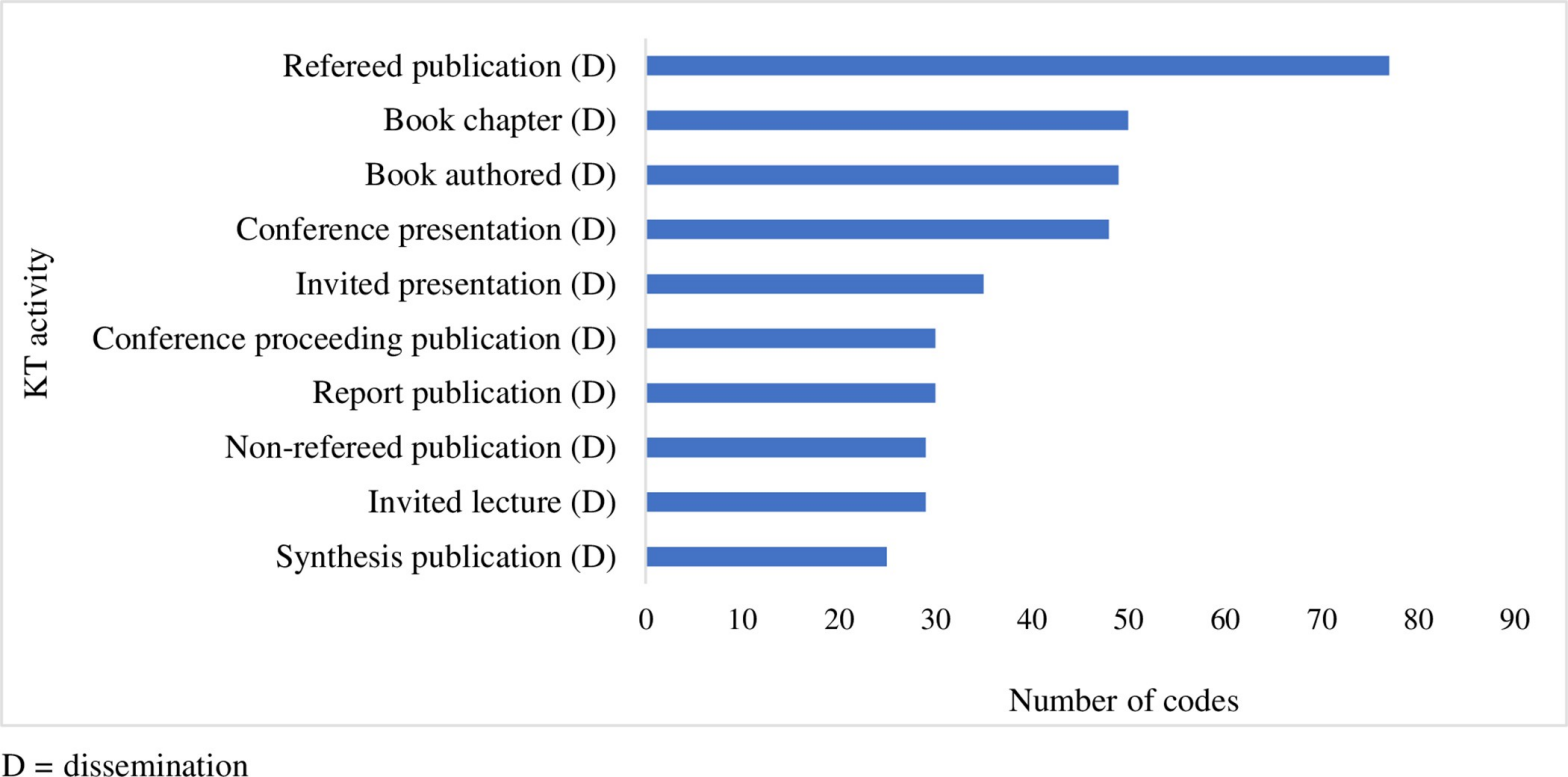
Ten most-commonly identified KT activities.

We identified 65 unique KT activities (range 0–60 activities/ document cluster, S3 Appendix). Most of the KT activities fell into the dissemination category (n = 42, 64.6% of unique activities), followed by application (n = 8, 12.3%), synthesis (n = 8, 12.3%) and exchange (n = 7, 10.8%). The 10 most-commonly identified KT activities, nine of which are categorized as dissemination activities, are presented in Fig 2.

### Latent KT content

We identified two latent categories: primarily implicit recognition of KT; and an overall lack of clarity for KT.

#### Implicit recognition of knowledge translation in T&P documentation

We noted that KT was rarely addressed or discussed by name in the documents, rather that it was primarily recognized through the specific activities we identified as components of KT. For instance, only 16 of the 48 clusters included a specific reference to KT in a text segment with a total of 21 mentions, compared to 1038 text segments coded as a KT activity without referring to them as such. We interpreted this pattern as an implicit recognition of KT. Explicit, named recognition of KT was most prominent in health sciences-specific documents as opposed to institution-level documents (20 of the 21 mentions), although the language used was often brief (e.g. “knowledge transfer”, I-15, unit-level document), and did not use a comprehensive definition or fulsome explanation of what was considered as KT. Most documents that included explicit mention of KT were produced between 2015 and 2019 (8 of the 12 documents). In some cases, KT was referenced in relation to audiences outside of academic settings (e.g. “Outreach and Community Engagement may involve activities of knowledge translation to the non‐academic environment”, I-09, unit-level document).

#### Lack of clarity for knowledge translation

We also noted that most of the text segments we coded were short and contained minimal description or detail and were often provided in point form or within a list only. (e.g. “the application of knowledge”, I-08, institution-level document). We interpreted this as representing as lack of clarity for the reader (whether an applicant or reviewer), and as such would be unable to provide guidance on what would constitute a strong example of a KT activity or to meet a criterion to be considered as a specific activity. We noted that although the health sciences-specific documents contained more unique KT activities than institution-level documents (n = 43 in institution-level documents vs. n = 67 in health sciences-specific documents), they were not necessarily more detailed. We did identify a few exceptions to this lack of clarity in documentation, which stood out as noteworthy when they contained more information relative to the majority. For example, one instance from I-001, cluster 1:

*“Knowledge translation and impact* measured by:

- production of research outputs specifically designed to meet the needs of External Stakeholders, influence public health practice, public policy or lay audiences, technical contributions to improve research tools and instruments, commercialization activities; and/or
- evidence that the research helped to shape public health practice or public policy; and/or
- evidence that the research was utilized by External Stakeholders.”

## Discussion

Our analysis of 89 T&P institutional documents pertaining to 48 document clusters representing 100 health sciences academic units from 17 Canadian academic institutions demonstrated several key findings. First, we identified a clear emphasis on dissemination activities (over 80% of coded text segments and included in 47 of 48 document clusters). The most frequent dissemination activities represented traditional academic outputs (books, peer-reviewed publications, and academic conference presentations) with far less on activities targeted at knowledge users. Just over 15% of coded activities reflected any other KT category (application, exchange, or synthesis). This disproportionate distribution provides important signals as to what T&P documents codify as valued outputs in Canadian academic health sciences.

Second, the variation we identified in the nature and extent of KT recognition across document clusters was striking. The most comprehensive recognition of KT we found included nine documents in one cluster, which included reference to dozens of unique KT activities in all four categories. In contrast, some document clusters only included one document, and in a few cases the only document was an institution-level collective agreement, in which we found no mention of KT. Overall, the lack of explicit KT recognition and clarity we identified has implications for providing guidance to T&P applicants and reviewers. Our findings could be supplemented in future research with faculty members and T&P decision-makers to more fulsomely examine how KT activities are implicitly considered in academic advancement decision-making.

While our study was the first to examine T&P considerations from the lens of KT, our work partially replicates existing findings that showed an emphasis on traditional T&P criteria in 15 Canadian research-intensive faculties of medicine [42]. Given the partial overlap in sampling between their analysis and our present study, this shared finding is understandable. However, their criteria were limited to dissemination activities whereas our analysis examines the full scope of KT. The unique contribution of our analysis lies in our consideration of application and synthesis activities in academic work and their role in T&P considerations. We note that despite general enthusiasm for evolved concepts of scholarship and adoption by some health sciences disciplines through structures such as clinical accreditation [53], formal adoption of these concepts in Canadian academic health sciences advancement structures has been sub-optimal.

The primarily implicit recognition of KT activities in Canadian health sciences institutional T&P documents suggests that Canadian universities currently do not formally encourage an explicit and comprehensive approach to KT. Implicit recognition, in combination with the lack of clarity and guidance, may perpetuate limited KT practices. The focus on dissemination activities emphasizes a limited subset of KT practices that are least likely to have societal impacts. We note that many of the noted dissemination activities do address non-academic audiences (such as broadcast, print, or social media), reflecting some recognition of efforts to move beyond peer audiences. However, some of the dissemination activities reflect smaller, time-limited, discrete tasks (such as a conference presentation, or abstract publication), whereas other KT activities noted as a single line item in consideration for T&P may represent comprehensive bodies of work that can require years to complete and significant resource investments (e.g. systematic reviews, clinical practice guidelines). The continued emphasis on a limited range of dissemination activities reflects a habitual emphasis on traditional outcomes that have been encultured as long-standing expectations of faculty, a finding also identified by others [43].

It is not clear from these documents whether the vague KT considerations in Canadian T&P documents reflect a lack of understanding of KT, a deliberate attempt to be inclusive (letting any and all activities be considered in T&P processes), or a deliberate effort to not value the role of KT in scholarship. The lack of specificity does permit a degree of flexibility–while not necessarily encouraging explicit KT work, faculty are equally not discouraged from doing it. Despite the potential advantages of flexibility inherent in T&P documentation, the overwhelming emphasis on dissemination and traditional academic outputs may serve to disincentivize and devalue more intensive yet potentially impactful KT work. What these documents do suggest is a general low priority for comprehensive KT in academic health sciences that collectively reinforce and perpetuate the question: what is valued by academic institutions?

We acknowledge several limitations of our approach. Our sample was restricted to primarily research-intensive Canadian academic institutions, so the findings may not be generalizable to universities that are not research intensive. Our document search was restricted to publicly-available documents or those which institutions chose to share with us; as such we cannot verify if these represent all of the relevant documents for each health sciences academic unit. We did not validate the document cluster groupings with institutions. We recognize that our analysis only focused on official documentation, and additional guidance about inclusion of KT activities may be provided through oral communication or in other formats (e.g., presentations) to which we did not have access. We can only comment on written documentation, which does not mean T&P decisions strictly follow these guidelines, therefore findings will not be conclusive about KT recognition in overall T&P processes. Lastly, we acknowledge the challenges associated with evolving conceptualizations of KT which are inherently linked with applying contemporary thinking retrospectively.

In conclusion, our analysis showed vague considerations of KT in Canadian academic health sciences institutional T&P documents, supporting previous research highlighting concerns about the recognition of KT work as a barrier to its practice [29–31, 34, 39]. Having noted the importance of these documents as structural drivers of the relationship between research and society, when left vague, they reinforce status quo. We recommend that academic institutions consider and address the value of KT activities, and where appropriate, align documentation policies and procedures to reflect these values. Development of tools for supporting KT, such as institutional KT strategic plans (e.g. [54]), along with specific guidance for applicants and reviewers, may help to address both structural and individual barriers limiting academic KT work. Without further resources and supports, the application KT activities will be left to individual academics developing their T&P applications and/ or reviewers. Furthermore, we call on the innovative spirit of health researchers to continue to challenge traditional views of scholarship and embrace the diverse practices inherent in the work of KT to have meaningful impact within and beyond academic boundaries. To ensure this analysis reaches academic decision makers, we will share the findings and key recommendations with all participating institutions and associated units, develop a social media strategy, and disseminate to relevant target audiences (i.e. U15 Group of Canadian Research Universities, Canadian Association of Research Administrators, Universities Canada). We will share the findings with our networks and leadership tables that leverage the distributed networks we are connected to, with an invitation to generate dialogue about implications of these findings.

## Supporting information

S1 Appendix Coding framework. (DOCX)

S2 Appendix Included institutions and health sciences disciplines. (DOCX)

S3 Appendix Unique KT activities. (DOCX)
