# Supplementary material for: Recognition of knowledge translation practice in Canadian health sciences tenure and promotion: A content analysis of institutional policy documents
Source: PLoS One. 2022 Nov 17;17(11):e0276586. doi: 10.1371/journal.pone.0276586 (PMC9671374; doi:10.1371/journal.pone.0276586)
Supplement: S1 Appendix — (DOCX) [file pone.0276586.s001.docx]

**Appendix 1: Coding Framework**

1. Institution characteristics

- Institution name?
- Institution type? (U15 or non-U15)
- Province of institution?
- Faculty unionized or not?

1. Document characteristics

- Document version year?
- Document access date?
- Document URL?
- Document title?
- Document type? (collective agreement, policy, guideline, template)
- Document level? (institution-level document or faculty-level document)
- Faculty level? (faculty, school, or college)

1. KT activities reported

| **Explicit KT recognition in document** | **KT recognition type** | **Specific KT component** | **KT activity** |
| --- | --- | --- | --- |
| - Yes  - No | - Specific  - Generic | **Synthesis**  The contextualization and integration of research findings of individual research studies within the larger body of knowledge on the topic. | - Scoping review - Systematic review - Overview of reviews - Review of reviews - Meta-analysis - Network review - Literature review - Meta-narrative synthesis/review - Realist review - Rapid review - Other: - Not specified |
|  |  | **Dissemination**  Identifying the appropriate audience and tailoring the message and medium to the audience. | - Journal article - Books - Monographs - Conference presentations (poster/ oral) - Educational materials - Events/courses - Interactive small group meeting - Plain-language summaries - Policy briefs - Summary briefings - Reminders - Social media activities - Media release/outreach campaign - Arts-based activity - Web-based activities (e.g. postings, wikis, blogs, podcasts, etc.) - Handbooks - Infographics - Other: - Not specified |
|  |  | **Exchange**  Interactions between knowledge users and researchers resulting in mutual learning. | - Partnerships - Outreach - Community engagement - iKT - Engaged scholarship - Knowledge broker involvement - Communities of practice - Participatory research - Collaboration - Action-oriented research - Participatory action research - Community-based research - Other: - Not specified |
|  |  | **Application**  The iterative process by which knowledge is put into practice. | - Implementation - Patent - Clinical practice guidelines - Adoption - Use - Uptake - Commercialization - Other: - Not specified |

All definitions are taken from: http://www.cihr-irsc.gc.ca/e/29418.html
