# Supplementary material for: Recognition of knowledge translation practice in Canadian health sciences tenure and promotion: A content analysis of institutional policy documents
Source: PLoS One. 2022 Nov 17;17(11):e0276586. doi: 10.1371/journal.pone.0276586 (PMC9671374; doi:10.1371/journal.pone.0276586)
Supplement: S2 Appendix — (DOCX) [file pone.0276586.s002.docx]

**Appendix 2: Included institutions and health sciences disciplines**

| **Province** | **Institution** | **Faculty** | **Cluster** |
| --- | --- | --- | --- |
| Newfoundland Labrador | 1. Memorial University | 1.1 Faculty of Medicine | X |
|  |  | 1.2 Faculty of Nursing  1.3 School of Pharmacy  1.4 School of Human Kinetics & Recreation | X |
| Prince Edward Island | 2. University of Prince Edward Island | 2.1 Faculty of Nursing | X |
| Nova Scotia | 3. Dalhousie University | 3.1 Faculty of Health  3.2 School of Health & Human Performance  3.3 School of Health Administration  3.4 School of Health Sciences  3.5 School of Communication Sciences & Disorders  3.6 School of Nursing  3.7 School of Occupational Therapy  3.8 School of Physiotherapy  3.9 College of Pharmacy | X |
|  |  | 3.10 Faculty of Medicine | X |
|  |  | 3.11 Faculty of Dentistry | X |
| New Brunswick | 4. University of New Brunswick | 4.1 Faculty of Kinesiology  4.2 Faculty of Nursing | X |
| Quebec | 5. McGill University | 5.1 Faculty of Medicine & Health Sciences  5.2 Faculty of Dentistry  5.3 Ingram School of Nursing  5.4 School of Communication Sciences & Disorders  5.5 School of Physical & Occupation Therapy  5.6 School of Population & Global Health | X |
|  | 6. University of Montreal | 6.1 Faculty of Pharmacy | X |
|  |  | 6.2 Faculty of Medicine | X |
|  |  | 6.3 School of Rehabilitation  6.4 School of Speech-Language Pathology & Audiology  6.5 Faculty of Dentistry  6.6 Faculty of Nursing  6.7 School of Public Health  6.8 School of Optometry | X |
|  | 7. Laval University | 7.1 Faculty of Dentistry  7.2 Faculty of Medicine  7.3 Faculty of Pharmacy  7.4 Faculty of Nursing | Not able to access documents |
| Ontario | 8. McMaster University | 8.1 Faculty of Health Sciences  8.2 School of Nursing  8.3 School of Rehabilitation Science  8.4 Michael G. DeGroote School of Medicine | X |
|  | 9. University of Ottawa | 9.1 Faculty of Medicine | X |
|  |  | 9.2 Faculty of Health Sciences  9.3 School of Human Kinetics  9.4 Interdisciplinary School of Health Sciences  9.5 School of Nutrition Sciences  9.6 School Rehabilitation Sciences  9.7 School of Nursing | X |
|  |  | 9.8 School of Epidemiology & Public Health | X |
|  | 10. Queens University | 10.1 Faculty of Health Sciences  10.2 School of Medicine  10.3 School of Nursing  10.4 School of Rehabilitation Therapy | X |
|  |  | 10.5 School of Kinesiology & Health Studies | X |
|  | 11. University of Toronto | 11.1 Faculty of Kinesiology & Physical Education | X |
|  |  | 11.2 Dalla Lana School of Public Health | X |
|  |  | 11.3 Faculty of Dentistry | X |
|  |  | 11.4 Leslie Dan Faculty of Pharmacy | X |
|  |  | 11.5 Temerty Faculty of Medicine | X |
|  |  | 11.6 Lawrence S. Bloomberg Faculty of Nursing | X |
|  | 12. University of Waterloo | 12.1 Faculty of Applied Health Sciences (now Faculty of Health) | X |
|  |  | 12.2 School of Optometry & Vision Science | X |
|  |  | 12.3 School of Pharmacy | X |
|  |  | 12.4 School of Public Health & Health Systems (now School of Public Health Sciences) | X |
|  | 13. Western University | 13.1 Schulich School of Medicine & Dentistry | X |
|  |  | 13.2 Faculty of Health Sciences  13.3 School of Communication Sciences & Disorders  13.4 School of Health Studies  13.5 School of Kinesiology  13.6 Arthur Labatt Family School of Nursing  13.7 School of Occupational Therapy  13.8 School of Physical Therapy | X |
| Manitoba | 14. University of Manitoba | 14.1 Rady Faculty of Health Sciences  14.2 Max Rady College of Medicine  14.3 College of Rehabilitation Sciences | X |
|  |  | 14.4 Dr. Gerald Niznick College of Dentistry  14.5 School of Dental Hygiene | X |
|  |  | 14.6 College of Pharmacy | X |
|  |  | 14.7 College of Nursing | X |
|  |  | 14.8 Faculty of Kinesiology & Recreation Management | X |
| Saskatchewan | 15. University of Saskatchewan | 15.1 College of Medicine | X |
|  |  | 15.2 College of Dentistry | X |
|  |  | 15.3 School of Rehabilitation Science | X |
|  |  | 15.4 College of Nursing | X |
|  |  | 15.5 College of Kinesiology  15.6 College of Pharmacy & Nutrition  15.7 School of Public Health | X |
| Alberta | 16. University of Alberta | 16.1 School of Public Health | X |
|  |  | 16.2 Faculty of Medicine & Dentistry | X |
|  |  | 16.3 Faculty of Nursing | X |
|  |  | 16.4 Faculty of Pharmacy & Pharmaceutical Sciences  16.5 Faculty of Rehabilitation Medicine  16.6 Faculty of Kinesiology, Sport, and Recreation | X |
|  | 17. University of Calgary | 17.1 Cumming School of Medicine | X |
|  |  | 17.2 Faculty of Kinesiology | X |
|  |  | 17.3 Faculty of Nursing | X |
| British Columbia | 18. University of British Columbia | 18.1 Faculty of Medicine | X |
|  |  | 18.2 Faculty of Dentistry  18.3 School of Population & Public Health  18.4 Faculty of Pharmaceutical Sciences  18.5 Faculty of Health & Social Development  18.6 School of Kinesiology  18.7 School of Health & Exercises Sciences  18.8 School of Nursing  18.9 School of Audiology & Speech Sciences | X |
