# Supplementary material for: Recognition of knowledge translation practice in Canadian health sciences tenure and promotion: A content analysis of institutional policy documents
Source: PLoS One. 2022 Nov 17;17(11):e0276586. doi: 10.1371/journal.pone.0276586 (PMC9671374; doi:10.1371/journal.pone.0276586)
Supplement: S3 Appendix — (DOCX) [file pone.0276586.s003.docx]

**Appendix 3:** **Unique KT activities**

|  | **Activity category** | **Coded activities included** |
| --- | --- | --- |
| **Dissemination** | | |
| 1 | Abstract presentation |  |
| 2 | Book authored | Books authored, Monographs |
| 3 | Book chapter |  |
| 4 | Book edited |  |
| 5 | Book review |  |
| 6 | Broadcast media dissemination | Radio, TV, media productions |
| 7 | Brochure |  |
| 8 | Case study publication | Case report publications, Case review publications, Case series, Case study publications |
| 9 | Citation publication |  |
| 10 | CME CDP Presentation |  |
| 11 | Commercialization |  |
| 12 | Conference presentation | Conference presentations, Generic presentations, Refereed presentations |
| 13 | Conference proceeding publication | Abstract publications, Publishing conference proceedings |
| 14 | Database publication |  |
| 15 | Dissemination to lay people | Dissemination to community organizations, dissemination to lay people, publications for lay people, Public/ patient education |
| 16 | Editorial |  |
| 17 | Educational publication | Continuing education texts, Encyclopedia entries, Mededportal, Mededpublish, Textbooks, manual publications, reference texts |
| 18 | Electronic publication | Electronic publications, non-print publications, online resources |
| 19 | Forum | Community forum, consensus forum, policy forum |
| 20 | Grand rounds |  |
| 21 | Guest speaker |  |
| 22 | Guideline publication |  |
| 23 | Invited lecture |  |
| 24 | Invited presentation |  |
| 25 | Invited publication |  |
| 26 | Keynote presentation |  |
| 27 | Media interview | Media interviews, media appearances |
| 28 | Mortality rounds |  |
| 29 | Multimedia dissemination | Digital storytelling, CDs, Choreography, films, screenings, recordings, videos/audios, Vodcasts, Software/applications |
| 30 | Non-refereed publication |  |
| 31 | Opinion/commentary publication | Views publication, Invited commentary, Letters to journals/editors, Reflective texts |
| 32 | Oral presentation |  |
| 33 | Pamphlet |  |
| 34 | Panel/ seminar speaker | Panels, seminars |
| 35 | Policy paper/report | Briefs, Policy papers, Policy reports |
| 36 | Poster presentation |  |
| 37 | Presentation to government/community agencies |  |
| 38 | Print media dissemination | Transcripts or tapes of broadcast material, Columns, Magazines, newsletters, newspaper articles |
| 39 | Public presentation |  |
| 40 | Refereed publication | Refereed publications, research articles, generic dissemination, generic publications |
| 41 | Report publication | General reports, White papers, Research reports, Scholarly reports, Technical reports, Expert consensus statements |
| 42 | Sign-in rounds |  |
| 43 | Social media dissemination | Social media dissemination, Twitter, Youtube |
| 44 | Symposium |  |
| 45 | Synthesis publication | Literature review publications, Publishing reviews |
| 46 | Teaching rounds |  |
| 47 | Web-based dissemination | Podcosts, Blogs, Contributions to academic websites, Webpages, Websites, |
| 48 | Webinar |  |
| 49 | Workshop |  |
| **Exchange** | | |
| 1 | Community/stakeholder engagement | Community engagement, Engagement with community members, Engagement with stakeholders, engagement with media |
| 2 | Engaged scholarship |  |
| 3 | Interdisciplinary collaborations | Interdisciplinary collaborations , Collaboration with researchers |
| 4 | Knowledge mobilization |  |
| 5 | Participatory action research |  |
| 6 | Partnership | Partnership with industry, , Partnership with policy/decision makers, Partnership with service providers, , Partnership with community organizations, Partnership with government agencies |
| 7 | Research collaboration |  |
| **Synthesis** | | |
| 1 | Conducting reviews |  |
| 2 | Knowledge synthesis |  |
| 3 | Practice guidelines |  |
| 4 | Practice standards |  |
| 5 | Scholarship of integration |  |
| 6 | Tool development |  |
| **Application** | | |
| 1 | Application of knowledge |  |
| 2 | Applied scholarship/ research | |
| 3 | Implementation of programs/resources | |
| 4 | Patents |  |
| 5 | Quality improvement |  |
| 6 | Research impact |  |
| 7 | Scholarship of application |  |
| 8 | Technology transfer |  |
